# Microclimate prediction for sandy photovoltaic power plants using a spatio-temporal graph convolutional network with environmental covariates

**DOI:** 10.1016/j.isci.2026.116329

**Published:** 2026-06-11

**Authors:** Jianjun Li, Zekun Yang, Weiyi Wang, Haoran Li, Weifeng Dong, Bo Zhou, Mingda Liu, Ming Li

**Affiliations:** 1 School of Energy and Transportation Engineering, Inner Mongolia Agricultural University, Hohhot 010000, China; 2 Huaneng New Energy Co., Ltd., Mengxi Branch, Hohhot 010000, China

**Keywords:** environmental science, applied sciences, artificial intelligence

## Abstract

The deployment of photovoltaic (PV) power plants in sandy regions alters the local microclimate. Accurate microclimate prediction is essential for optimizing short-term operations and maintenance plans. Compared with traditional statistical and numerical models, neural networks are suited to capture the complex nonlinear dependencies in sandy-region PV environments. Graph neural networks (GNNs) extend neural networks to process data defined on graph domains, enabling them to confront high-dimensional feature redundancy, attenuated temporal dependencies, and pronounced spatial heterogeneity. Accordingly, we propose a spatio-temporal graph convolutional network with environmental covariates (EC-STGCN) for station-level microclimate prediction at meteorological stations. Experimental results demonstrate that EC-STGCN achieves accurate high-resolution spatio-temporal predictions of temperature, relative humidity (RH), and particulate matter (PM_10_) and shows improved predictive performance relative to conventional GNNs, while covariate-level analysis shows that individual environmental covariates contribute heterogeneously to these predictions.

## Highlights

• Minute-ahead microclimate forecasting for sandy-region PV plants
• Environmental covariates reduced errors over 10–60 min horizons
• Spatiotemporal graph models with environmental covariates outperformed baselines
• The model provides guidance for vegetation management and sand control in sandy PV

## Graphical abstract

**Figure:**
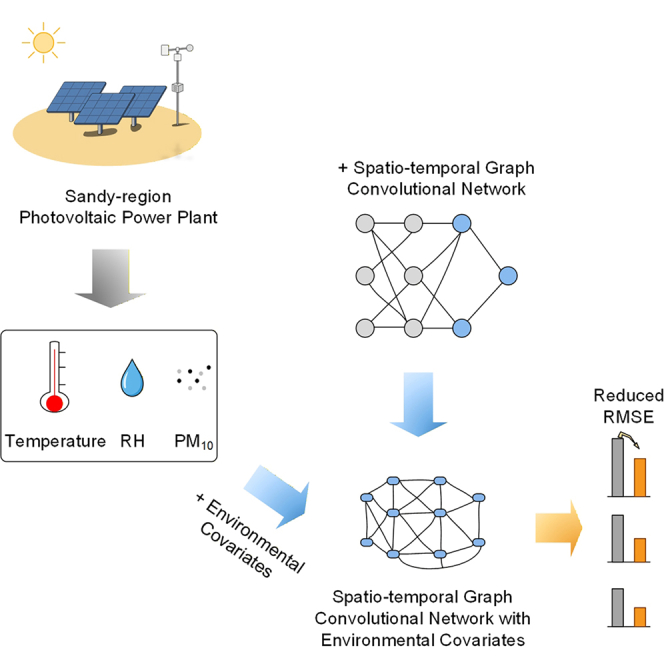

## Introduction

Over the past decade, as the global energy system has accelerated its transition, photovoltaic (PV) systems have been widely deployed in desertification control projects owing to their low carbon footprint and flexible deployment.^1^ In arid and semiarid regions, large-scale PV plants have promoted ecological rehabilitation, wind and sand mitigation, and efficient land use, and assessments across multiple scales indicate that desert PV systems have demonstrated net positive effects on ecosystems, and reasonable predictions offer useful guidance for management decisions.^2^^,^^3^^,^^4^ Meanwhile, PV arrays alter surface radiative fluxes, thermal distributions, and wind fields, thereby markedly influencing local microclimates, and microclimatic changes directly affect module performance and vegetation survival.^5^^,^^6^

Barron-Gafford et al. reported that night-time temperatures under a semiarid PV plant were 3°C–4°C higher than those in surrounding open land.^7^ Vervloesem et al. analyzed the functional diversity of vegetation across different locations and observed that shade-tolerant and moisture-tolerant functional groups concentrated in shaded zones.^8^ When panels are sufficiently elevated, the shading effect can substantially reduce surface temperature, potentially facilitating vegetation growth.

PV plants deployed on rugged, non-graded sandy terrain may exhibit elevation contrasts and local topographic variability, which can produce systematic inter-station differences in radiative exposure and near-surface airflow.^9^^,^^10^ Vegetation within PV plants may also be actively managed through drought-tolerant planting, resulting in a relatively stable and spatially structured surface state at seasonal timescales. Accordingly, the normalized difference vegetation index (NDVI) serves as a proxy for surface and vegetation state.^11^ Sand-barrier type reflects roughness and sheltering effects relevant to mixing and dust resuspension.^12^ Terrain descriptors and geographic coordinates provide quasi-static spatial context and background gradients that are difficult to disentangle from temporal variability alone.^13^ Incorporating environmental covariates with meteorological sequences introduce complementary spatial context and persistent surface-state information. This synergy allows the model to reconcile long-term background regimes with transient short-term dynamics, thereby enhancing its predictive robustness and cross-site generalization.

For microclimate prediction, a range of statistical and machine learning methods have been applied to forecast near-surface meteorological variables. It is critical for many disciplines to obtain continuous spatio-temporal data with high resolution.^14^ Guo et al.^15^ employed a hybrid model based on seasonal autoregressive integrated moving average, cointegration, and error correction, which incorporates historical air temperature, dew point temperature, humidity, precipitation, and other covariates to forecast relative humidity (RH). However, these models usually treat environmental covariates merely as additional input features. They do not explicitly model environmental heterogeneity via spatial adjacency or graph structure. In desert PV plants, short-term dust events and fluctuations in RH can rapidly alter soiling accumulation and dust-moisture cementation, thereby enhancing particle adhesion and rendering cleaning effectiveness highly sensitive to timing.^16^^,^^17^^,^^18^ Meanwhile, rapid changes in irradiance, ambient temperature, and RH largely affect module operating temperature and associated thermal-stress conditions.^19^ Many prior forecasting studies operate at hourly or coarser temporal resolutions across both urban and field settings,^20^^,^^21^^,^^22^ which constrains their ability to capture high-frequency dynamics and inter-variable interactions.

This limitation reduces their applicability in complex desert PV settings where spatial heterogeneity is important. These constraints have motivated growing interest in spatio-temporal graph network models. Graph-based models provide a framework for capturing spatial heterogeneity by representing monitoring sites as nodes and their interactions as edges.^23^^,^^24^ In contrast to traditional approaches that impose grid-based Euclidean assumptions, graph representations accommodate irregularly distributed station networks with non-Euclidean topology.^23^ By leveraging neighborhood aggregation, spatio-temporal graph networks explicitly model relational structures and learn inter-station dependencies beyond distance-based assumptions.^25^^,^^26^ This flexibility makes them well suited for modeling the highly heterogeneous environmental characteristics of desert PV plants.

High temporal resolution forecasts, therefore enable practical operations and maintenance (O&M) decisions by facilitating advance planning and resource mobilization—such as pre-positioning cleaning resources and identifying cleaning windows—to minimize soiling-reduced revenue loss.^18^^,^^27^ They also provide early indications of thermal-stress risks under rapidly varying irradiance and ambient temperature.^19^ Additionally, such forecasts can guide vegetation and ground-cover maintenance planning, helping sustain the environmental co-benefits of maintaining vegetation within PV infrastructure.^2^

With the advent of graph-based methods, significant advances have been made in traffic flow and meteorological forecasting. Ma et al.^20^ proposed a hierarchical spatio-temporal graph neural network (GNN), which constructs a hierarchical graph via an adaptive graph learning module, achieving mean absolute error (MAE) and root mean square error (RMSE) reductions of 4.25% and 5.34% compared with the baseline, respectively. The microclimate vision model^21^ fuses street-level imagery with satellite data to predict air temperature and RH, reducing the RMSE of temperature and RH by 33.10% and 25.29% over the baseline, respectively. Wang et al.^22^ incorporated soil temperature and precipitation as external factors using GNN to capture the complex spatio-temporal dependencies, achieving MAE reductions of 49.53%, 34.86%, and 29.73% at three forecasting horizons, respectively. This multimodal approach can improve prediction accuracy compared with traditional observational models and offers methodological inspiration for PV microclimate prediction.

In this paper, we propose an enhanced spatio-temporal graph convolutional network (ST-GCN) framework, which constructs adjacency and edge weights based on environmental covariate similarity and geometric distance. This framework enables minute-level joint prediction of microclimate variables in desert PV settings, intending to support PV plant operation and vegetation management decisions.

## Related work

This section provides an overview of related research advances in four key aspects: the impact of microclimate data, commonly used prediction methods, concepts related to GNNs, and integration of environmental covariates.

### Methodological background

Microclimate conditions in sandy environments influence the performance of PV modules, where temperature, moisture, and vegetation cover jointly govern the near-surface hydrothermal regime.^28^^,^^29^ These variables also inform assessments of local ecological health and recovery.^1^ Accordingly, the site-level microclimate forecasting supports both PV O&M planning and ecosystem management.

**Figure 1:**
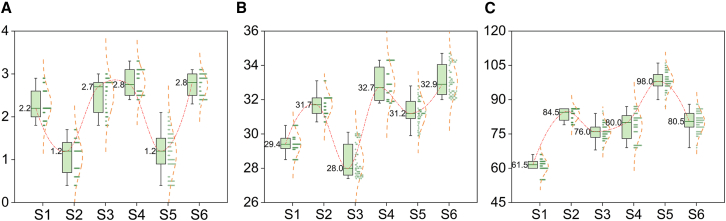
Distribution of meteorological variables at the monitoring stations (January 24, 2025, 14:00–14:59) (A) Temperature. (B) RH. (C) PM_10_.

However, unlike traditionally leveled PV plants, the preservation of original complex topography and vegetation in the study area significantly exacerbates the spatio-temporal heterogeneity of the environmental data. In these non-leveled terrains, the complex interplay between topography, vegetation, and other factors generates highly localized microclimatic variations.^1^^,^^2^^,^^28^ As evidenced by the diverse meteorological trends observed across different locations in Figure 1, traditional neural networks often struggle to capture the intricate dynamics of such complex scenes. Furthermore, although open-access datasets like ERA5 provide broad climate coverage, their coarse resolution is insufficient for resolving the fine-grained fluctuations within PV clusters.^30^^,^^31^ These deficiencies in data representativeness necessitate a robust modeling approach—such as the proposed ST-GCN—capable of leveraging non-Euclidean inter-site relational structures and heterogeneous covariates within a unified spatio-temporal framework.

Traditional microclimate studies often rely on numerical weather prediction models.^32^ Classical machine learning methods, such as Bayesian neural networks, linear regression, and support vector machines, have commonly been employed to predict irradiance. With the advent of deep learning, the nonlinear modeling capability of many models has significantly improved in handling large-scale, high-dimensional meteorological data. Convolutional neural networks (CNNs), which are sensitive to local spatial features, have been widely used to predict wind speed and urban heat islands, achieving high accuracy.

However, traditional weather station data are often interpolated into raster format before model input. The interpolation process may introduce cumulative errors and distort the original signal characteristics, which in turn affect the subsequent modeling results.

In contrast, graph-based models overcome the aforementioned limitations by directly processing non-Euclidean data and have made breakthroughs in graph tasks such as social networking, traffic flow, and air quality. In the field of transportation, the temporal graph convolutional network (T-GCN) model has successfully achieved traffic flow prediction by integrating gated recurrent unit (GRU) with graph convolution,^26^ which provides a methodological basis for weather prediction. In the scenario of weather forecasting, researchers adopted similar approaches by training an adaptive adjacency matrix to predict meteorological variables across multiple stations. However, the learned adjacency matrix is highly sensitive to initial parameters and thus less stable. To address this, some scholars combined CNNs, attention mechanisms, and climate context embedding to construct models for precipitation prediction.^33^ Another category of ST-GCN models has demonstrated strong predictive performance on real-world traffic flow datasets from two distinct regions through the integration of graph convolutional network (GCN) and temporal convolutional network (TCN).^25^ Compared with previous work, this paper incorporates environmental factors while considering the spatio-temporal nature of different weather stations in a sandy environment, thereby enabling more accurate modeling of complex weather phenomena.

### GNNs

**Figure 2:**
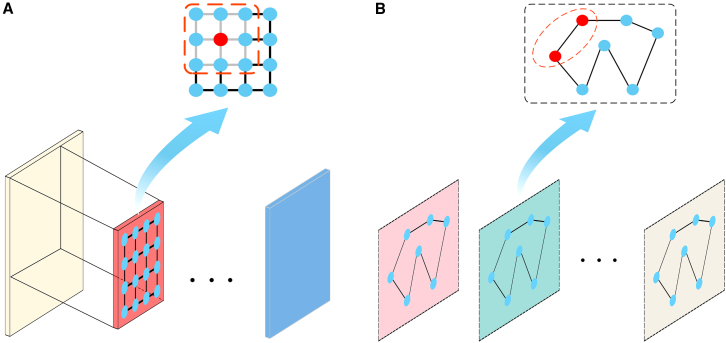
Comparison between 2D convolution and graph convolution (A) 2D convolution. Each pixel in the image is treated as a node, and the domain is determined by the filter size. (B) Graph convolution. In contrast to image data, the neighbors of graph nodes are unordered and differ in quantity.

GNNs are a class of deep learning frameworks specifically designed for non-Euclidean data structures. The classical GNN was proposed in 2009, with the core idea of learning node features and topological relationships through iterative information propagation.^24^ As illustrated in Figure 2, two-dimensional convolution can be interpreted as domain aggregation over regular grids, whereas graph convolution extends this concept to irregular topologies.

The classical GNN framework comprises two core steps: message passing and state updating.^34^ During message passing, each node aggregates information from its neighbors to update its own state, and after repeated iterations, the hidden states of nodes converges. Compared with traditional CNNs and recurrent neural networks (RNNs), GNNs offer significant advantages when handling data with irregular topologies, naturally enabling feature extraction and analysis of non-Euclidean spatial data segments.

Time-series forecasting is widely used in fields such as transportation, finance, electricity, and meteorology.^35^^,^^36^ Machine learning-based methods utilize *T* steps of historical data to predict *P* steps of future target data and have demonstrated strong performance in capturing periodicity, trends, and dependency analysis.^37^ However, meteorological variables have significant spatial correlation. In rugged sandy environments, the geographic location differences between stations can significantly affect the prediction results. Spatial information is one of the key factors in improving model accuracy, but when dealing with multi-site data, traditional time-series models often fail to capture the spatial dependence among multiple sites simultaneously.^23^

The ST-GCN-based models treat monitoring stations as graph nodes to capture spatial dependencies^20^ and construct adjacency matrices based on metadata such as geographic location, elevation, and other features, thereby explicitly modeling spatial dependencies.^38^

Although ST-GNNs couple temporal modeling with graph message passing, they often rely on fixed or purely data-driven graph structures that may be mismatched for stations deployed over complex terrain. In such settings, interaction strength is expected to decay with geographic separation and terrain differences. We therefore learn adaptive edge weights to regulate message passing, mitigating spatial mismatch, and reducing the risk of over-smoothing.

### Incorporation of environmental covariates

Gupta et al.^39^ relied on geo-referenced measurements, spatially collocated the samples with gridded covariates, and extracted covariate values at the sampling locations, thereby enabling cross-regional spatial prediction. Similarly, Htitiou et al.^40^ derived phenological metrics that characterize seasonal vegetation dynamics from time-series remote sensing data and incorporated topographic and climatic covariates to complement spatially continuous background information. These studies indicate that collocating point observations with covariates encoding terrain, vegetation, and climate can better represent spatial heterogeneity and support model transferability.

Among indicators describing vegetation growth status, NDVI has been used to assess biomass dynamics and vegetation distribution.^41^^,^^42^ In arid regions, NDVI serves as a proxy for land-surface hydrothermal conditions and vegetation condition.^43^^,^^44^ Stress covariates constructed from meteorological data and process-based models have further been shown to improve prediction accuracy in unobserved environments and reduce prediction variability.^45^

Building on this evidence, this study incorporates NDVI, elevation, spatial location descriptors (latitude and longitude) and sand-barrier type as environmental covariates in desert-PV microclimate forecasting. These covariates are jointly modeled with meteorological time series to characterize how land-surface conditions and terrain structure modulate microclimate evolution.

## Results

### Comparison with GNNs benchmarks

**Figure 3:**
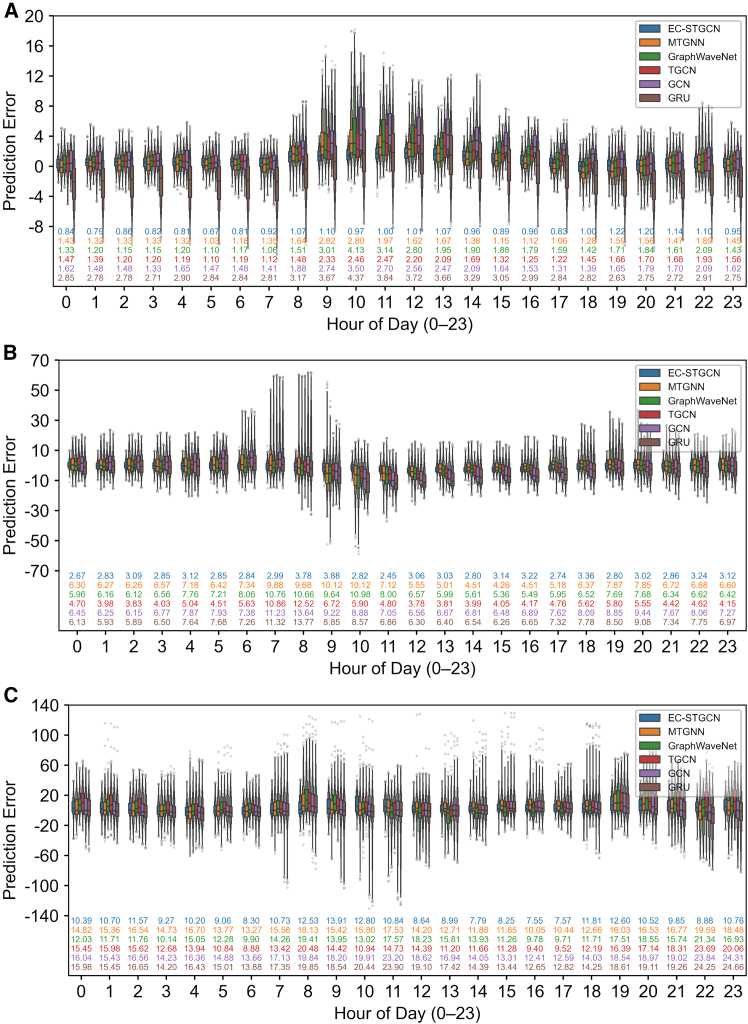
Comparison between EC-STGCN and GNN baselines (A) Temperature prediction error of models (°C) and error SD. (B) RH prediction error of models (%) and error SD. (C) PM_10_ prediction error of models (μg/m^3^) and error SD. EC-STGCN, spatio-temporal graph convolutional network with environmental covariates; GCN, graph convolutional network; MTGNN, multivariate time series graph neural network; GRU, gated recurrent unit.

Given the diurnal variability of microclimate, we conducted an hour-of-day stratified statistical summary of the test dataset results (hours 0–23), aggregated across all stations. As illustrated in Figure 3, the hierarchical grouped boxplots use distinct colors to represent different models, with the thick central portion of each box indicating the interquartile range (IQR, middle 50% of the data). Figures 3A–3C display the hourly error distributions for temperature, RH, and PM_10_, respectively. In addition, we annotate the standard deviation (SD) of the errors at each hour (with numeric values color-matched to the corresponding model) to provide a quantitative measure of error dispersion.

For temperature (Figure 3A), the baselines show noticeably wider error distributions between 09:00 and 15:00, whereas EC-STGCN maintains consistently low dispersion throughout the day (SD: 0.67°C–1.22°C). During these periods, the SD of EC-STGCN remains lower than that of the other models, demonstrating effective suppression of diurnal error fluctuations. For RH (Figure 3B), EC-STGCN achieves an SD range of 2.45%–3.88% and a diurnal mean SD of 3.02%. Relative to TGCN, which has the smallest dispersion among the baselines and a diurnal mean SD of 5.30%, EC-STGCN reduces dispersion by 43.02%. Error variability is particularly pronounced in the 08:00–10:00 window, where the mean SD values are 3.49 and 8.38 for our model and TGCN, respectively, corresponding to a 58.35% reduction. For PM_10_ (Figure 3C), EC-STGCN exhibits shorter boxes and fewer outliers across the same periods. Its diurnal mean SD is 10.15 μg/m^3^, corresponding to a 28.67% reduction compared to Graph WaveNet, the least-dispersive baseline, with a diurnal mean SD of 14.23 μg/m^3^. Moreover, the baseline models show wider error distributions, more pronounced long tails, and more concentrated outliers between 07:00 and 13:00. Over the same interval, the mean SD of EC-STGCN is 30.11% lower than that of Graph WaveNet. Overall, EC-STGCN shows lower error dispersion than the baselines. Its mean SD is 0.96°C for temperature, 3.02% for RH, and 10.15 μg/m^3^ for PM_10_, compared with the best baseline dispersions of 1.53°C (MTGNN), 5.30% (TGCN), and 14.23 μg/m^3^ (Graph WaveNet), and dispersion is reduced by 37.25%, 43.02%, and 28.67%, respectively.

Error fluctuations are driven by raw data—the greater the variability, the harder accurate prediction becomes. The occurrence of large magnitude fluctuations is common in sandy environments, as natural conditions such as solar radiation and rainfall, as well as anthropogenic factors like vegetation and PV layout, can cause substantial instability in microclimate data. By integrating environmental covariates, the model mechanistically captures variation patterns of microclimate in sandy-area PV power plants across different periods.

Table 1 summarizes the comparative prediction performance of the proposed model and four classical graph-based models and a time series model GRU on the microclimate dataset from the sandy-area PV power station, with the best results highlighted in bold. The RMSE, MAE, and coefficient of determination (R^2^) values presented in the table are averaged across the six monitoring stations. Compared with the plain GCN baseline, EC-STGCN achieves substantial performance improvements. After incorporating environmental covariates, the errors for all three meteorological variables on the EC-STGCN model decrease significantly: the temperature RMSE drops from 3.59°C to 1.40°C, the RMSE for RH decreases from 9.41% to 3.31%, and the RMSE for PM_10_ is reduced from 17.76 μg/m^3^ to 11.12 μg/m^3^—reductions of 61.00%, 64.82%, and 37.39%, respectively. Meanwhile, R^2^ increases from 76.83%, 90.60%, and 77.27% to 97.19%, 98.31%, and 88.29%, respectively. Although the errors increase with larger forecasting horizons, the proposed model consistently outperforms all baselines.

**Table 1:** Comparative performance of each model on 30 min test data (averaged across sites)

Model	Temperature			RH			PM_10_		
	RMSE	MAE	R^2^	RMSE	MAE	R^2^	RMSE	MAE	R^2^
GRU	3.59	2.84	76.83	9.41	6.96	90.60	17.76	12.68	77.27
GCN	3.17	2.38	86.34	9.17	6.81	91.21	17.68	12.51	77.51
TGCN	2.33	1.72	91.25	6.32	4.45	93.92	16.75	11.63	75.27
Graph WaveNet	2.63	1.82	88.57	7.86	5.69	92.48	16.26	11.73	72.57
MTGNN	2.13	1.51	91.22	7.46	5.34	93.10	15.84	11.48	78.51
EC-STGCN	1.40	1.11	97.19	3.31	2.66	98.31	11.12	8.13	88.29

R^2^ is reported on a percentage scale (0%–100%) and is used consistently throughout the paper.

As evidenced by the aggregated results in Table 1, EC-STGCN systematically exceeds the performance of all baseline models. This suggests that environmental covariates offer complementary predictive information that improves prediction accuracy. In the subsequent sections, we further analyze the impact of individual covariates on model performance from both temporal and spatial perspectives.

The experimental results demonstrate that EC-STGCN consistently outperforms baseline models across all meteorological variables, particularly in reducing intra-day error fluctuations. These improvements suggest that incorporating environmental covariates contributes significantly to prediction stability.^46^ In contrast to baseline models, EC-STGCN explicitly incorporates surface heterogeneity through environmental covariates, resulting in more stable predictions across diurnal cycles.

### Time performance analysis

**Figure 4:**
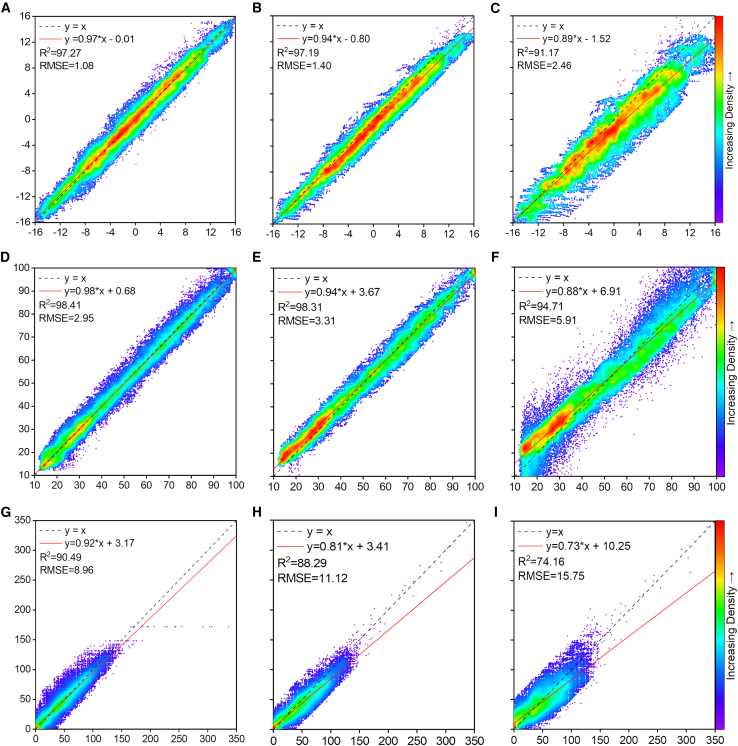
Density scatter plots for multiple variables across three forecast horizons; the solid and dashed lines denote the ideal line (*y = x*) and the regression line Panels are organized by variable and forecast horizon: (A–C) temperature, (D–F) RH, and (G–I) PM_10._ Within each row, the columns correspond to the 10-, 30-, and 60-min forecast horizons, respectively. The x- and y-axes represent the observed and predicted values, respectively.

In microclimate forecasting, prediction error accumulates as the lead time increases. In this subsection, we analyze forecasts from all weather stations using linear regression and scatter-density plots. Figure 4 exhibits model predictions with observations for temperature, RH, and PM_10_ at 10, 30, and 60 min. The abscissa represents observation, the ordinate represents the prediction, and the color of scatter point indicates density.

Errors for all three variables increase monotonically as the horizon lengthens. Vertically, the model performs best at 10 min: scatterplots for temperature, RH, and PM_10_ are closely aligned with the ideal line *y* = *x* with R^2^ reaching 97.27%, 98.41%, and 90.49% respectively, indicating that the model effectively captures short-term fluctuations. Extending to 30 min, RMSE rises by 29.6%, 12.2%, and 24.1% for temperature, RH, and PM_10_, respectively. Although overall prediction accuracy remains acceptable at this scale, the PM_10_ scatter points begin to deviate from *y* = *x*. When the forecast horizon is extended to 60 min, errors for all variables increase significantly: the RMSE values rise to 2.46°C, 5.91%, and 15.75 μg/m^3^ for temperature, RH, and PM_10_, respectively—representing increases of 1.06°C, 2.60%, and 4.63 μg/m^3^ compared to the 30-min horizon. The scatter bands also become visibly wider. PM_10_ is most affected, with the R^2^ dropping to 74.16%, the RMSE increasing to 15.75 μg/m^3^, and the regression line deviating markedly from the ideal reference *y* = *x*.

In summary, prediction errors for all three meteorological variables increase steadily as the forecast horizon lengthens. The marked long-horizon uncertainty in particulate matter (PM_10_) is tied to the inherently complex, variable microclimate of sandy environments.

As shown in Table 2, extending the forecast horizon from 10 min to 60 min leads to a systematic degradation in model performance. For temperature, RMSE rises from 1.08°C to 2.46°C, MAE increases from 0.84°C to 1.89°C, and R^2^ falls from 97.27% to 90.49%. RH exhibits a similar downward trend. PM_10_ is the most sensitive to forecast length: RMSE increases from 8.96 μg/m^3^ to 15.75 μg/m^3^, MAE from 6.27 μg/m^3^ to 10.95 μg/m^3^, and R^2^ drops by 19.4%.

**Table 2:** RMSE, MAE, and R^2^ of meteorological variables at different forecast horizons

	RMSE			MAE			R^2^		
	10 min	30 min	60 min	10 min	30 min	60 min	10 min	30 min	60 min
Temp	1.08	1.40	2.46	0.84	1.11	1.89	97.27	97.19	91.17
RH	2.95	3.31	5.91	2.26	2.66	4.89	98.41	98.31	94.71
PM_10_	8.96	11.12	15.75	6.27	8.13	10.95	90.49	88.29	74.16

The differential impact of covariates across horizons reflects their temporal relevance.^47^ Prediction errors for all three meteorological variables increase steadily as the forecast horizon lengthens. PM_10_ is particularly sensitive to long-horizon forecasting, likely due to its dependence on transient meteorological conditions and land surface dynamics. These findings suggest that future models may benefit from integrating dynamic covariates—such as wind speed and solar radiation—to better capture particulate variability over extended time scales.

### Spatial performance analysis

**Figure 5:**
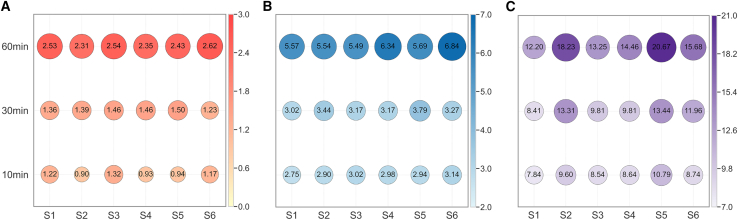
Spatial performance is summarized using bubble charts. Bubble size and color encode RMSE, and a label centered in each bubble reports its RMSE value (A) Temperature RMSE. (B) RH RMSE. (C) PM_10_ RMSE.

Figure 5 summarizes the RMSE distribution of EC-STGCN across six stations for 10, 30, and 60 min horizons. Across the 60-min horizon, temperature and RH show small absolute RMSE changes (|ΔRMSE| < 5) and broadly similar error growth across stations (Figures 5A and 5B). In contrast, PM_10_ exhibits pronounced spatial divergence, with the degree of error amplification differing substantially among sites at longer lead times (Figure 5C). Notably, the RMSE at S2 and S5 increases by 89.90% and 91.57%, respectively from 10 to 60 min. These represent the largest growth rates among the six stations, whereas growth rates at other sites remain below 80.00%. This uneven error amplification across stations indicates that, as the prediction horizon extends, spatial heterogeneity plays a more pronounced role in PM_10_ prediction performance.

This station-to-station variation is consistent with the site layout (Figure S1): S2 is located at the highest elevation where surrounding vegetation is sparse, while S5 lies near the site boundary adjacent to a PV-free zone. In both cases, sparse vegetation and nearby open areas likely lead to spatially varying near-surface dispersion and dilution conditions, favoring intermittent dust resuspension and short-range transport. By comparison, stations located deeper within denser PV rows and lush vegetation experience steadier near-surface dispersion and dilution, which can enhance particle retention and lead to smaller error variability.

Chang et al.^48^ showed that landform and vegetation patterns significantly shape microclimate dynamics in desert regions, which is consistent with the station-dependent divergence observed in PM_10_ predictions across stations. The reduced outliers and stabilized intra-day errors (Figure 3) improve the operational utility of short-term forecasts, supporting the scheduling of temperature-sensitive inspections and the dispatch of field crews. For PM_10_, improved short-term reliability supports proactive dust-risk readiness and helps prioritize soiling-related maintenance and vegetation protection.

### Ablation experiments

**Figure 6:**
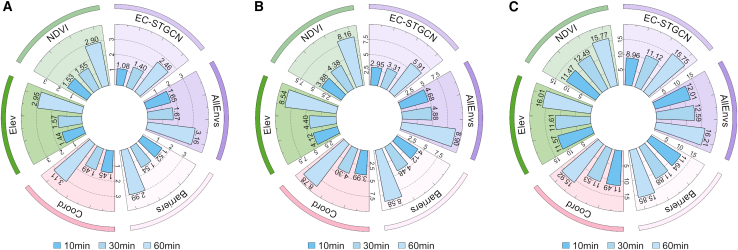
Radial bar charts of RMSE (A) Temperature. (B) RH. (C) PM_10_. Each sector corresponds to a model with the associated covariate removed; the three bars within each sector (clockwise) represent RMSE at 10, 30, and 60 min.

To quantify the marginal contributions of four environmental covariate categories, we performed an ablation study for temperature, RH, and PM_10_. In addition to the full EC-STGCN, we removed each covariate category individually and then removed all environmental covariates, yielding six experimental configurations, as summarized in Table 3. RMSE, MAE, and R^2^ for the 10, 30, and 60 min horizons are reported in Table S1 for temperature, Table S2 for RH, and Table S3 for PM_10_, with the corresponding RMSE trends visualized in Figure 6.

**Table 3:** Ablation strategy configuration

Strategy	Meaning
EC-STGCN	all environmental covariates
AllEnv	remove all environmental covariates, use only spatio-temporal features
Barriers	remove sand barrier type features
NDVI	remove NDVI features
Coord	remove latitude and longitude features
Elev	remove elevation feature

In the 10-min prediction window, EC-STGCN reduces the RMSE of temperature, RH, and PM_10_ by 34.55%, 36.97%, and 25.40%, respectively. The relative RMSE reductions of the three meteorological factors at 30 min are 16.17%, 32.17%, and 11.68%, respectively, indicating slightly diminished improvements compared to 10 min, yet still substantially outperforming the respective ablation models. For the 60 min long-term horizon, temperature and RH still achieve RMSE reductions of 22.15% and 33.60%, whereas the reduction for PM_10_ is limited to 2.85%. These results indicate that the long-range benefit of environmental information differs by variable and is least pronounced for PM_10_.

At all-time scales for all variables, EC-STGCN consistently yields the lowest error, while the AllEnv configuration produces the highest error. Figure 6 further substantiates this trend by visually illustrating the expansion of RMSE: each sector corresponds to the RMSE of a specific environmental covariate at 10-, 30-, and 60-min prediction horizons. As the prediction horizon lengthens, the marginal benefit of covariates declines, with the greatest gain achieved at 10 min. Because meteorological variables rely on different covariates, this behavior highlights distinct microclimate mechanisms. Future work will therefore explore adding dynamic covariates, such as wind speed and solar radiation, to enhance long-horizon performance.

Beyond the overall error reductions, Tables S1–S3 further show that the dominant environmental covariates shift with both the target variable and the forecast horizon. Results show that the primary drivers shift with both the target variable and the forecast horizon. For temperature, the dominant covariate shifts from NDVI at the 10-min horizon (removal increases RMSE by 29.41%) to elevation at 30 min (10.83% increase) and geographic coordinates at 60 min (20.90% increase). Similarly, RH predictability is initially governed by sand-barrier type, with removal causing RMSE increases of 28.40% at 10 min and 26.12% at 30 min. By the 60-min horizon, geographic coordinates emerge as the leading predictor, contributing RMSE increases of 32.69% for RH. Short-term PM_10_ forecasts are largely shaped by sand-barrier configuration and NDVI. In particular, omitting the sand-barrier type at the 10-min horizon results in a 23.02% increase in RMSE, while excluding NDVI at the 30-min horizon leads to a 10.97% rise in RMSE. As the forecasting horizon extends to 60 min, the contribution of each covariate drops to less than 1.63%.

## Discussion

The ablation study reveals that the predictive value of environmental covariates varies across temporal scales: NDVI and sand-barrier type contribute most to short-term forecasts, while static spatial features like latitude and longitude dominate at longer horizons. This differential impact reflects the temporal relevance of covariates. Temperature and RH are intrinsically coupled with land-surface properties and boundary-layer mixing—effectively captured by NDVI, sand-barrier configurations, and topographic descriptors—which provide robust station-specific constraints over short-term horizons. The diminishing utility of static covariates for long-horizon PM_10_ forecasting underscores the dominance of unmodeled temporal dynamics, such as transient wind shifts and stochastic dust events, which transcend the explanatory power of stationary environmental features. Amato et al.^49^ demonstrated that decomposing spatio-temporal processes into temporal bases and spatial coefficients enables flexible modeling of such dynamics, especially when covariate influence shifts over time. These findings point to the potential of dynamic covariate strategies in improving long-horizon particulate forecasts.

The ablation results indicate a horizon-dependent shift in covariate importance: the influence of localized land-surface heterogeneity (e.g., NDVI and sand-barrier type) decreases, whereas spatial differences among stations become more informative for prediction. This trend is apparent for both temperature and RH, where the dominant contributors shift from short-horizon surface descriptors to geographic coordinates at longer lead times, with coordinates ranking as the top contributor at the 60-min horizon for both variables. These results imply that longer-horizon forecasts depend increasingly on persistent spatial gradients encoded by station location, rather than on rapid, site-specific surface variability. For PM_10_, however, the long-horizon improvement remains limited, and the marginal gains from individual static covariates drop below 1.63%. In the future work, incorporating time-varying meteorological drivers may be necessary to improve extended-lead particulate forecasts.

The analysis shows that PM_10_ prediction errors exhibit clear diurnal peaks and outliers (notably at 7:00 and 9:00–13:00), stronger degradation at longer horizons, and pronounced inter-station divergence. This may be attributed to the fact that static environmental covariates capture spatial differences but are less effective in representing the dynamic and event-like variations of PM_10_, particularly at longer horizons. To mitigate the outliers and long-horizon degradation observed for PM_10_, the model can be refined by: (1) incorporating sand-transport observations, (2) deriving additional surface and terrain descriptors from DEM, such as slope and roughness, and (3) labeling dust events for event-aware training and evaluation.

Taken together, the experimental results show that environmental covariates enhance the performance of EC-STGCN, particularly for temperature and RH, whereas PM_10_ remains more difficult to forecast, reflecting its stronger dependence on complex transport and dispersion dynamics. Addressing this limitation may require the incorporation of time-varying meteorological drivers, sand-transport descriptors, and dust-event information into extended-lead particulate forecasting.

At the framework level, EC-STGCN integrates temporal dependencies, spatial interactions, and environmental context to deliver more stable predictions. Mechanistically, the improved stability arises because node-level covariates (NDVI and sand-barrier type) stabilize station regimes through gated fusion, while edge attributes (horizontal distance and elevation difference) produce learned edge weights that modulate message passing and reduce over-smoothing. This integrated model offers quantitative support for module operation and maintenance, thermal mitigation strategies, and ecological protection, while also emphasizing the value of combining meteorological time series with ecological and engineering covariates to improve practical applicability and transferability.

### Limitations of the study

Despite the demonstrated potential of integrating environmental covariates with ST-GCN for microclimate prediction in sandy PV power plants, several limitations remain. NDVI, sand-barrier type, and geographic coordinates provide useful station-specific constraints but their static nature may limit their ability to capture highly transient processes, such as abrupt wind shifts, episodic dust fluctuations, and short-term sand transport dynamics. This limitation affects long-horizon forecasting of PM_10_, where transient episodic dynamics may outweigh stationary environmental controls. Future work could help mitigate this issue by incorporating time-varying meteorological drivers and event-aware indicators reflecting abrupt environmental changes. It could also explore transformer-based temporal modules or dynamic graph structures to better capture evolving inter-station dependencies.

The limited spatial and temporal coverage of the observations may restrict the generalizability of the findings. The dataset was collected at a single sandy PV power plant over nearly 5 months (autumn to winter) and may not fully capture the annual cycle of desert microclimatic variability. Future multi-site studies could examine variations in PV array configurations, such as panel tilt, row spacing, and mounting height, as well as terrain and vegetation differences, to evaluate the framework’s performance across different sandy PV power plants. Beyond inter-site differences, detailed observations of microscale heterogeneity within PV arrays—such as under-panel and inter-row zones—could help clarify localized impacts of shading and near-surface heat exchange. The sparse and uneven distribution of monitoring stations across sand-barrier types may hinder separating sand-barrier effects from site-specific characteristics and complicate the assessment of sensitivity to station density. Establishing larger scale, multi-site monitoring networks would further enhance the robustness and practical utility of the framework.

## Resource availability

### Lead contact

Further information and requests for resources and reagents should be directed to and will be fulfilled by the lead contact, Ming Li (nylm@imau.edu.cn).

### Materials availability

This study did not generate new unique reagents.

### Data and code availability

• The source code supporting the implementation of the model are publicly available at: https://github.com/RushVon/EC-STGCN.
• The complete raw and processed monitoring datasets are not publicly available due to project regulations and institutional data-use policies. Requests for access to the data for non-commercial academic purposes should be directed to the lead contact, Jianjun Li (jianjun.lee@outlook.com), and will be evaluated in accordance with relevant institutional and project-related policies. Data may be made available subject to approval and the completion of a formal data use agreement.
• Any additional information required to reanalyze the data reported in this article is available from the lead contact upon reasonable request.

## Acknowledgments

This research was funded by the 10.13039/501100004763Natural Science Foundation of Inner Mongolia Autonomous Region under grant 2024LHMS03069, in part by the Basic Research Project of Basic Scientific Research Business Fees in Universities Directly through the Inner Mongolia Autonomous Region under grant BR220139, and in part by the Technology Research Project of Huaneng Group under grant HNKJ24-H116.

## Author contributions

Methodology, data curation, formal analysis, software, validation, visualization, and writing – original draft, J.L.; investigation, J.L., Z.Y., W.W., and H.L.; writing – review and editing: J.L., Z.Y., and M.Li; conceptualization, funding acquisition, and supervision, M.Li; project administration, W.D., B.Z., and M.Liu; resources, W.D., B.Z., and M.Liu.

## Declaration of interests

The authors declare no competing interests.

## STAR★Methods

### Key resources table

**Table:** 

REAGENT or RESOURCE	SOURCE	IDENTIFIER
**Software and algorithms**		

Python 3.10	Anaconda	https://www.anaconda.com/
NumPy 1.26	pypi	https://pypi.org/project/numpy/1.26.4/
Pandas 1.5	pypi	https://pypi.org/project/pandas/1.5.0/
Scikit-learn 1.5	pypi	https://pypi.org/project/scikit-learn/1.5.1/
PyTorch 2.2.2	PyTorch	https://pytorch.org/get-started/previous-versions/
CUDA 12.1	NVIDIA	https://developer.nvidia.com/cuda-12-1-0-download-archive
PyTorch Geometric 2.5.2	PyG	https://pytorch-geometric.readthedocs.io/en/2.5.2/install/installation.html

**Deposited data**		

Data	This paper	https://github.com/RushVon/EC-STGCN.git

### Experimental model and study participant details

Omitted as our study does not involve biological models.

### Method details

A detailed description of the dataset is provided in the Data collection and preprocessing section, and the experiment was conducted on a workstation with the following specifications: OS – Windows 11 Pro, CPU – Intel Xeon Gold 6430 @ 2.10 GHz, RAM – 256 GB, GPU – RTX A6000.

#### Study area

The Bayannur region of Inner Mongolia is characterized by low annual precipitation and high evaporation, which makes it a key area both for the implementation of the Three-North Shelterbelt Program and for regional desertification control.^50^ The study site is a photovoltaic power station located within the Xin Hua State Forest Farm in Linhe District, Bayannur (41°02′ N, 107°35′ E). The area is a typical desertified landscape, sparsely covered with large and dwarf shrubs. The surface sediments are predominantly sandy, loosely packed, and exhibit weak inter-particle cementation.

In recent years, regional desertification control programs have adopted a comprehensive strategy comprising engineered sand fixation, artificial shrub planting, and grass seeding. With the development and stabilization of planted vegetation in the conservation area, the surface sand shifted from a loose to a semi-fixed condition. This transition altered key near-surface microclimatic variables, particularly wind speed and ground temperature.^51^ Six ground-based meteorological stations were installed within the photovoltaic plant (Figure S1), which covers an area of approximately 115 m × 275 m. The station’s construction and management have enhanced the local ecological environment, aligning with the Inner Mongolia Autonomous Region’s policy to integrate desertification control with wind and solar photovoltaic development. These unique conditions render the site a representative case for microclimate studies and provide a robust observational basis for evaluating the impacts of desert-based photovoltaic installations on the near-surface microclimate.

#### Data collection and preprocessing

This study employed two categories of data: meteorological observations and environmental covariates. Meteorological data were used for model construction and performance evaluation, while environmental covariates characterized the underlying surface and spatial context and were incorporated into the model as inputs. Station 1 in the north lies in a sandbag barrier zone, Station 2 in the center is in a reed-mat barrier zone, Station 3–Station 6 in the south are in rice-straw barrier zones. Data were collected over 140 days (21 October 2024 to 9 March 2025), with measurements recorded at 1-min intervals, yielding 201,600 records per variable at each station. The PV station is deployed on natural sandy terrain that has not been leveled or graded. The monitoring stations are distributed across the study area with an elevation relief of 15 m (min: 974 m, max: 989 m).

Sensors were RS-BYH-M louvered transmitters (Table S4), mounted at heights of 2.0 m and 2.5 m above the ground, in compliance with the Chinese national standard GB/T 42477–2023. To minimize bias from solar-panel reflection and localized heating, all sensors were installed on the rear side of the panels.^52^ Data are transmitted in real time via a 4G network to a cloud platform, enabling continuous remote monitoring.

This study uses meteorological observations and environmental covariates collected at the study site. The dataset was split into training, validation, and test subsets in a strict chronological order with an 8:1:1 ratio. The earliest segment of the time series was assigned to training, followed sequentially by validation and testing, ensuring temporal independence between datasets and preventing potential information leakage from future data. We evaluated model performance for prediction horizons of 10, 30, and 60 min by using different historical input lengths *T*, to compare prediction accuracy and robustness across time horizons.

The analysis considered four environmental covariates: NDVI, sand-barrier type, geographic coordinates (latitude and longitude), and elevation. These covariates were selected to provide physically interpretable, spatially explicit boundary-condition information and to remain static or slowly varying over the prediction horizon, thereby helping explain persistent inter-station heterogeneity.^53^^,^^54^

The factors such as PV tilt angle, under-panel soil type, and wind speed were not included because some factors such as PV panel tilt angle are fixed and exhibit little station-scale variability in our study area, while others such as wind speed are partly modulated by local terrain and surface roughness and may therefore be redundant with the selected surface-context descriptors, increasing the risk of multicollinearity.

NDVI is adopted as a dynamic indicator for vegetation vigor^41^ and was derived from Unmanned Aerial Vehicle (UAV) imagery collected with 85% sidelap and frontlap, and it is updated every two months. Sand-barrier type, geographic coordinates, and elevation were obtained through field surveys and UAV orthophoto processing, and are considered static site attributes. Sand barriers are engineered sand-stabilization measures that modify near-surface roughness and airflow.^55^ Inter-station geographic distance derived from latitude and longitude was used to construct graph connectivity between stations.^53^

In combination, these covariates effectively reflect the key underlying surface features provide complementary environmental context describing vegetation condition, engineered surface roughness, and site location and topographic height of the desert PV site and provide essential environmental context to the prediction model for the model.^53^^,^^54^^,^^56^

To ensure the temporal and spatial consistency of the model inputs, meteorological and environmental data were preprocessed, and key features were extracted. The meteorological data were first processed to handle missing and abnormal values. Missing observations were filled using multiple interpolation methods, and outliers in temperature, RH, and PM_10_ were then identified using the boxplot method and replaced with the mean of neighboring time points. All variables were then resampled to a uniform 1-min resolution and timestamps were synchronized across stations to ensure temporal alignment.

Radiometric correction was applied to the UAV multispectral imagery, after which reflectance values in the near-infrared (NIR) and red (R) bands were extracted to compute the NDVI, defined as $NDVI=\frac{NIR-R}{NIR+R}$. Each monitoring station served as the center of a circular sampling area, and all pixels within a 1 m radius (corresponding to the 2 m × 2 m sand-barrier grid) were averaged to derive the site-level NDVI indicator. Sand barrier types were encoded using one-hot vectors, while latitude, longitude, and elevation were extracted from orthophotos produced by UAV image mosaicking. These static spatial features were used to calculate inter-site horizontal distances and elevation differences, which were then integrated into the model’s weighted adjacency matrix.

#### Model design

The EC-STGCN was developed as an integrated framework to model the coupled temporal dynamics, spatial dependencies, and environmental heterogeneity in desert PV microclimates. Its architecture comprises three interdependent modules: temporal encoding, spatial graph convolution, and environmental feature fusion (Figure S2), which capture multiscale dependencies and integrate heterogeneous information for microclimate prediction. The inputs to the model are the meteorological tensor *X* and the environmental covariates *e*, as shown in Equation 1 and Equation 2.

(Equation 1) $$\begin{array}{c} X=\left[x_{1},x_{2},\cdots ,x_{T-1},x_{T}\right],x_{t}\in R^{N\times M}\; \end{array}\text{,}$$

(Equation 2) $$\begin{array}{c} e=\left[e_{1},e_{2},\cdots ,e_{N}\right],e_{i}={[e}_{i}^{S},e_{i}^{NDVI},e_{i}^{A},e_{i}^{La},e_{i}^{Lo}], \end{array}$$

where T is the historical sequence length, N is the number of stations (*N* = 6), and M is the number of meteorological variables (M = 3).

For each station i, the historical meteorological sequence in *X* is encoded by a BiGRU, the forward and backward hidden states at the final time step are concatenated to yield the temporal embedding *h*_*i*_∈*R*^2*H*^, where H denotes the hidden dimension. The temporal embeddings of *N* stations are stacked to form *H*_*temporal*_∈*R*^*N*×2*H*^, which serves as the unified temporal representation. This bidirectional configuration captures both past and future contextual information, mitigates the vanishing gradient, and improves the modeling of nonlinear smoothing processes typically observed during nocturnal microclimate transitions.

Having obtained temporal embeddings *h*_*i*_, environmental information is divided into two categories: node-level covariates (NDVI, sand-barrier type) and spatial metadata (coordinates, elevation, sensor height). Spatial metadata is used to construct the graph topology and edge attributes, while node-level covariates are reserved for later feature fusion.

NDVI is treated as a time-varying node covariate and embedded by a two-layer MLP applied to the last time step. Sand-barrier type is one-hot encoded and mapped to a low-dimensional embedding via a two-layer MLP, which is used as a node-level feature and fused with spatio-temporal representations for prediction.

(Equation 3) $$\begin{array}{c} g_{ij}=\left[d_{ij},\Delta e_{ij}^{A},{sens}_{i},{sens}_{j},e_{i}^{A},e_{j}^{A}\right], \end{array}$$

where $e_{i}^{A}$ and $e_{j}^{A}$ denote the elevation, *sens*_*i*_ and *sens*_*j*_ denote the sensor height and are retained for completeness of the spatial edge representation. For each station pair (i, j), the horizontal distance *d*_*ij*_ is computed from ($e_{i}^{La},e_{i}^{Lo}$) and ($e_{j}^{La},e_{j}^{Lo}$) using the haversine formula, and the elevation difference is defined as $\Delta e_{ij}^{A}=e_{i}^{A}-e_{j}^{A}$. A spatial edge is created only when *d*_*ij*_≤*τ*, where *τ* is a predefined distance threshold, resulting in an adjacency structure.

The edge feature *g*_*ij*_ is encoded by a multilayer perceptron to produce edge attributes, which are symmetrized. The unnormalized *α*_*ij*_ is an unnormalized edge score produced by *MLP*_*edge*_, whose parameters are learnable.

(Equation 4) $$\begin{array}{c} \alpha_{ij}=MLP_{edge}\left(g_{ij}\right), \end{array}$$

The edge scores are then normalized to obtain learned edge weights *w*_*ij*_, which are used as weights in graph convolution:

(Equation 5) $$\begin{array}{c} w_{ij}=\frac{\exp\left(\alpha_{ij}\right)}{\sum_{k=1}^{N}\exp\left(\alpha_{ik}\right)\;} \end{array}\text{,}$$

The learned edge weights *w*_*ij*_ define a weighted adjacency *W*, which is used in subsequent graph convolution. Self-loops are excluded by default and are introduced only when no valid inter-station edges can be constructed. Given *H*_*temporal*_ and *W*, a multi-layer residual GCN is applied to model spatial interactions, producing the spatial representation *H*_*spatio*_, which encodes node-level features learned through message passing.

After spatial graph convolution, the model incorporates local environmental context through an environmental feature fusion module. NDVI and sand-barrier type are encoded and fused with spatio-temporal node representations, whereas elevation is incorporated into the edge attributes. Geographic coordinates are used to compute inter-station distances for spatial graph construction and edge attributes. The fusion module takes *H*_*spatio*_, temporal embeddings *h*_*i*_, and encoded environmental covariates as inputs and outputs fused node representations for prediction. A regression head is then applied to forecast surface temperature, RH, and PM_10_. The model is trained end-to-end using a weighted MSE loss, incorporating L_1_ and L_2_ regularization as well as temporal smoothness and consistency terms (L_S_, L_Temp_). Early stopping based on validation metrics is used to mitigate overfitting.

Unlike traditional feature fusion stacks multiple feature maps along the channel dimension, EC-STGCN employs an attention-based gating mechanism for the adaptive fusion of features to suppress noise signals across different sites and forecast horizons. To address irregular topography, the model learns physics-driven adaptive edge weights directly from geospatial attributes to regulate message-passing intensity based on these physical priors. The framework mitigates spatial mismatch and over-smoothing risks typically associated with fixed adjacency structures. Finally, the model adopts a decoupled spatio-temporal paradigm, in which intra-station temporal encoding precedes inter-station message passing. This design alleviates noise in minute-level high-frequency sequences, thereby enhancing both numerical stability and predictive accuracy.

#### Graph construction and input preprocessing

Graph topology and edge-weight learning follow Equations 3, 4, and 5. Each node corresponds to a meteorological station. For each station pair (i, j), the horizontal distance *d*_*ij*_ is computed between stations using the Haversine formula. We create spatial edges if *d*_*ij*_≤*τ* (τ=1000m). In our station layout, all distance satisfies this threshold, thus forming a complete graph with no isolated nodes. Self-loops are excluded by default and are only introduced as a safety fallback when no valid inter-station edges can be constructed.

Edge features are fed to the model in raw physical units. Edge scores *α*_*ij*_ are produced by a two-layer MLP, *α*_*ij*_ = *MLP*_*edge*_(*g*_*ij*_), and then normalized to obtain edge weights W = w_ij_.

The meteorological tensor *X* in Equation 1 includes M = 3 variables: temperature, RH, and PM_10_. The historical sequence length is T, and the model performs multi-step forecasting with a forecast horizon of P steps. Sliding windows are constructed with a stride of 1.

Environmental covariates *e* in Equation 2 include node-level covariates (NDVI and sand-barrier type) and spatial metadata (coordinates, elevation, sensor height). NDVI is treated as a time-varying node covariate and encoded by a two-layer MLP applied to the NDVI value at the last time step, sand-barrier type is one-hot encoded and mapped to a low-dimensional embedding via a two-layer MLP for feature fusion.

To avoid data leakage, all scalers are fitted on the training split only and then applied to validation and test sets. Specifically, temperature, RH, and PM_10_ are standardized using StandardScaler, while NDVI is normalized using MinMaxScaler.

#### Model architecture and feature fusion

The temporal encoder is a two-layer bidirectional GRU with a hidden dimension of 128 and a dropout rate of 0.35. The spatial module consists of a three-layer residual graph convolutional network, equipped with residual connections, LayerNorm and BatchNorm, and GELU activations. Environmental information is incorporated via a FeatureFusion module using four-head attention, gating, and an MLP, with a dropout rate of 0.30. Finally, a two-layer MLP regression head maps the fused representations to an output tensor of size P × 3.

The FeatureFusion module integrates four per-station feature streams: temporal features from the BiGRU, spatial representations from the GCN, static sand-barrier embeddings, and NDVI embeddings. All streams are projected into a shared H-dimensional latent space (with the temporal stream reduced from 2H to H) and are reshaped to batch × N × H. The temporal and spatial streams are then refined by four-head self-attention with residual connections and LayerNorm. A learnable 4 × H gating matrix, normalized by a softmax across the four streams, assigns channel-wise weights before concatenation. The weighted streams are concatenated (4H) and fused by a two-layer MLP (4H→2H→H) with LayerNorm, GELU, and dropout = 0.3, producing the final fused representation used by the regression head.

#### Model training

The data are split chronologically into the training, validation, test sets in an 8:1:1 ratio. Model parameters are optimized using AdamW with a learning rate of 1 × 10^−4^ and weight decay of 1 × 10^−3^. Training runs for up to 400 epochs with a batch size of 16, gradient clipping at 0.5, and mixed-precision training enabled. The learning rate schedule adopts a 10-epoch warmup followed by cosine decay to a minimum learning rate of 1 × 10^−6^.

The training objective is a weighted mean squared error with additional regularization and consistency terms:

(Equation 6) $$\begin{array}{c} L=L_{wMSE}+\lambda_{1}L_{1}+2\lambda_{2}L_{2}+\lambda_{s}L_{smooth}+\lambda_{t}L_{temp,} \end{array}$$

with *λ*_1_ = 1 × 10^−4^, *λ*_2_ = 1 × 10^−3^, *λ*_*s*_ = 0.05, and *λ*_*t*_ = 0.008. Dynamic per-target loss reweighting is performed with an update interval of K = 5. The best checkpoint is selected based on the lowest validation loss. Here L_wMSE_ denotes the weighted MSE over the three target variables, where per-target weights are dynamically adjusted during training every K updates. L_1_ and L_2_ regularize the learnable feature-weight vector used in L_wMSE_. L_smooth_ penalizes abrupt differences across adjacent predicted variables, while L_temp_ penalizes abrupt changes across consecutive forecast steps.

### Quantification and statistical analysis

#### Model evaluation

To assess the generalization and predictive performance of the proposed model, this study systematically evaluated the EC-STGCN and four baseline models under the same data-partitioning scheme. By training and testing all models using identical datasets, the fairness and reliability of the comparison are ensured.

Four representative GNNs—GCN, T-GCN, Graph WaveNet, and MTGNN—are selected as baselines for comparison with the proposed EC-STGCN. These baselines are widely recognized in the field of GNNs and have been extensively applied in spatio-temporal forecasting tasks such as traffic flow prediction and environmental modeling.

The evaluation framework consists of both quantitative and qualitative analysis. In the quantitative analysis, MAE, RMSE, R^2^ and SD are shown in Equations 7, 8, 9, and 10 are used to measure the deviation, fitting degree and dispersion between predicted and observed values. In the qualitative analysis, visual comparisons of model outputs and observations are conducted to illustrate performance differences across different temporal scales, spatial locations, and variables. By combining quantitative and qualitative evaluations, the predictive accuracy and generalization ability of the proposed model under multivariable and multiscale settings can be comprehensively assessed.

(Equation 7) $$\begin{array}{c} \text{MAE}=\frac{1}{n}\sum_{i=1}^{n}\left|Y_{i}-{\hat{Y}}_{i}\right| \end{array}\text{,}$$

(Equation 8) $$\begin{array}{c} \text{RMSE}=\sqrt{\text{MSE}}=\sqrt{\frac{1}{n}\sum_{i=1}^{n}{\left(Y_{i}-{\hat{Y}}_{i}\right)}^{2}}, \end{array}$$

(Equation 9) $$\begin{array}{c} R^{2}=1-\frac{\sum_{i=1}^{n}{\left(Y_{i}-{\hat{Y}}_{i}\right)}^{2}}{\sum_{i=1}^{n}{\left(Y_{i}-\bar{Y}\right)}^{2}}, \end{array}$$

(Equation 10) $$\begin{array}{c} \text{SD}=\sqrt{\frac{1}{n-1}\sum_{i=1}^{n}{\left(Y_{i}-{\bar{Y}}_{i}\right)}^{2}}\text{,} \end{array}$$

Where *Y*_*i*_ represents the observed value, ${\hat{Y}}_{i}$ denotes the predicted value, $\bar{Y}$ is the sample mean of the observed values, and n denotes the number of observations.

### Statistical analysis

In Figure 1, the boxplots and associated distribution summaries were generated in OriginPro 2024 using Box Normal. In Figure 4, the scatter plots and linear fitting results were obtained using Scatter plot and Linear fit in OriginPro 2024, respectively. MAE, RMSE, R^2^ and SD were calculated in Microsoft Excel 365 from paired predicted and observed values.
