# Supplementary material for: Microclimate prediction for sandy photovoltaic power plants using a spatio-temporal graph convolutional network with environmental covariates
Source: iScience. 2026 Jun 11;29(7):116329. doi: 10.1016/j.isci.2026.116329 (PMC13276428; doi:10.1016/j.isci.2026.116329)
Supplement: Document S1. Tables S1–S4 and Figures S1 and S2 [file mmc1.pdf]

iScience, Volume *29*

Supplemental information

Microclimate prediction for sandy photovoltaic power plants using a spatio-temporal graph convolutional network with environmental covariates

Jianjun Li, Zekun Yang, Weiyi Wang, Haoran Li, Weifeng Dong, Bo Zhou, Mingda Liu, and Ming Li

SUPPLEMENTAL INFORMATION

Ablation results for temperature, RH, and PM_{10}, with the best values highlighted in bold.

Table S1. Ablation in Temperature

Variable	Strategy	10min			30min			60min		
		RMSE	MAE	R^2	RMSE	MAE	R^2	RMSE	MAE	R^2
Temperature	AllEnv	1.65	1.19	95.27	1.67	1.20	95.05	3.16	2.44	85.98
	Barrier	1.52	1.09	96.48	1.54	1.17	95.86	2.99	2.30	86.89
	Coord	1.45	1.06	96.94	1.49	1.19	95.77	3.11	2.38	86.10
	Elev	1.44	1.13	97.05	1.57	1.20	95.59	2.95	2.24	85.92
	NDVI	1.53	1.12	97.07	1.55	1.19	95.88	2.90	2.17	86.78
	EC-STGCN	**1.08**	**0.84**	**97.27**	**1.40**	**1.11**	**97.19**	**2.46**	**1.89**	**91.17**

Table S2. Ablation in RH

Variable	Strategy	10min			30min			60min		
		RMSE	MAE	R^2	RMSE	MAE	R^2	RMSE	MAE	R^2
RH	AllEnv	4.68	3.28	96.93	4.88	3.59	96.35	8.90	7.21	90.82
	Barrier	4.12	3.26	97.85	4.48	3.27	97.16	8.58	6.92	91.51
	Coord	3.99	3.05	97.79	4.30	3.14	97.34	8.78	7.09	90.95
	Elev	4.12	3.15	97.77	4.40	3.23	97.38	8.54	6.80	91.15
	NDVI	3.88	2.89	97.86	4.38	3.22	97.35	8.16	6.39	91.47
	EC-STGCN	**2.95**	**2.26**	**98.41**	**3.31**	**2.66**	**98.31**	**5.91**	**4.89**	**94.71**

Table S3. Ablation in PM_{10}

Variable	Strategy	10min			30min			60min		
		RMSE	MAE	R^2	RMSE	MAE	R^2	RMSE	MAE	R^2
PM_{10}	AllEnv	12.01	8.62	85.94	12.59	9.60	84.65	16.21	11.58	71.05
	Barrier	11.64	8.09	86.13	11.88	9.04	85.57	15.85	11.08	71.88
	Coord	11.49	7.74	86.73	11.53	8.91	85.49	15.92	11.14	71.35
	Elev	11.57	7.87	86.71	11.61	8.90	85.48	16.01	11.21	70.83
	NDVI	11.47	7.91	87.12	12.49	9.47	83.84	15.77	11.18	71.93
	EC-STGCN	**8.96**	**6.27**	**90.49**	**11.12**	**8.13**	**88.29**	**15.75**	**10.95**	**74.16**

Table S4. Meteorological Sensor Specifications

Parameters	Range	Accuracy	Resolution
Temperature	−40 °C – −80 °C	± 0.5 °C	0.1 °C
RH	0 – 100%	± 3%	0.1%
PM_{10}	0 – 1000 μg/m³	± 10 μg/m³	1 μg/m³

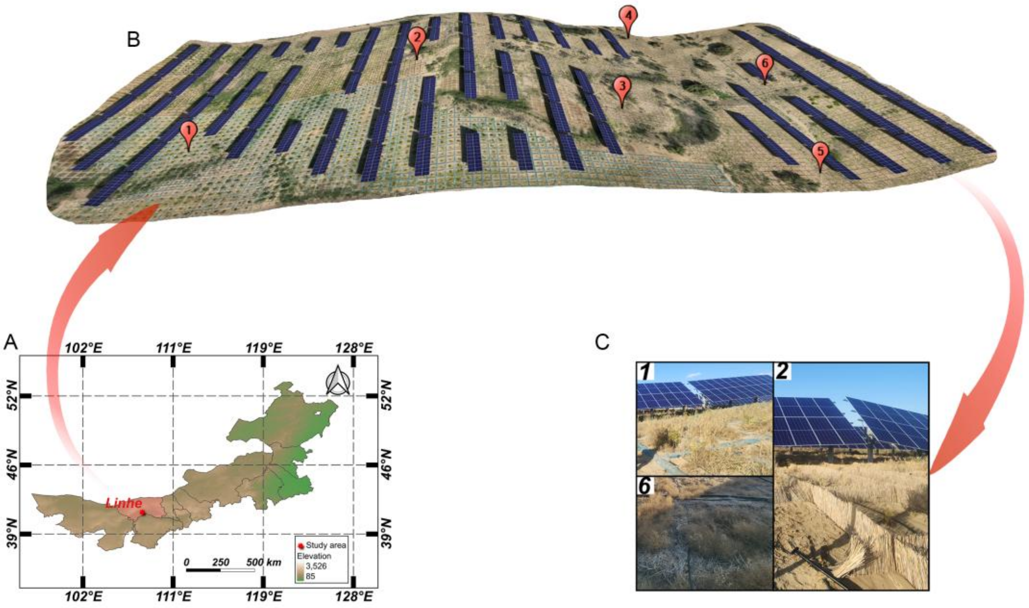

Figure S1. Study area and station locations used for model training

(A) Regional map of the Linhe District.

(B) Weather stations within the photovoltaic plant.

(C) Representative field photos illustrate the three sand-barrier types at three selected stations: Station 1 (sandbag barrier), Station 2 (reed-mat barrier), and Station 6 (rice-straw barrier).

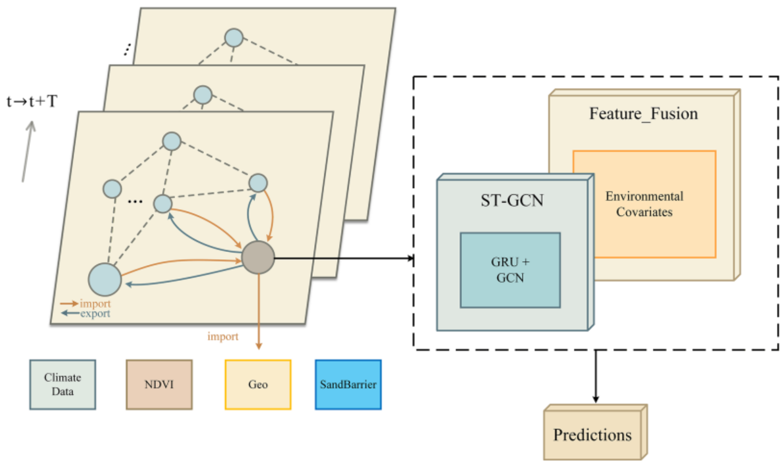

Figure S2. EC-STGCN architecture
